# Supplementary material for: RNA covariation at helix-level resolution for the identification of evolutionarily conserved RNA structure
Source: PLoS Comput Biol. 2023 Jul 14;19(7):e1011262. doi: 10.1371/journal.pcbi.1011262 (PMC10370758; doi:10.1371/journal.pcbi.1011262)
Supplement: Attachment — Submitted filename: response.pdf [file pcbi.1011262.s006.pdf]

June 2, 2023

Marc Robinson-Rechavi and Arne Elofsson
Editors
PLOSCB

Dear Dr. Robinson-Rechavi and Dr. Elofsson,

Thank you for the reviews of the manuscript “RNA covariation at helix-level resolution for the identification of evolutionarily conserved RNA structure” (PCOMPBIOL-D-23-00725). It is a treat to receive such nice reviews! And it has been a pleasure to work through all the well-informed points raised by the reviewers, which I have addressed as follows:

Reviewer #1

> The author argues provides evidence that the new helix aggregated metrics are more robust than the base-pair metrics. It would be helpful if they could provide suggestions or guidance to readers on what the roles of base pair vs helix level metrics should be: i.e., should each metric be calculated and assessed or are helix level metrics to replace base pair level ones? Are there cases where base pair level metrics could be better?

A very interesting comment that definitely deserves some space in the manuscript. I have added a paragraph in the Discussion section.

In summary, both helix and base-pair level covariation should be considered in combination. Consequently, R-scape reports them both superimposed in light and dark green respectively. The reason is twofold: on the one hand, we want to capture covariation from all possible structural elements including non Watson-Crick base pairs, and those do not arrange into helices as WC base pairs do. Also importantly, reporting helix-level covariation requires that a structure is proposed. This is ok when you know that you the RNA is structured, but it can be a source of artifacts when the structure is just a prediction, as I have shown in this paper as well. Base-pair level covariation on the other hand is agnostic to structure.

> When the author writes, “I observe that all methods except for Šidák report higher sensitivity for the same number of false positives than taking as supported all helices with at least one covarying base pair.” do they mean that the number of false positives doesn’t change with different values of k but sensitivity does, or that sensitivity divided by false positives grows?

The sentence is not clearly written. The number of false positives gets smaller as the k-unaggregated increases (so does the sensitivity). The point is that for a given k with a particular number of false positives, the aggregated methods (except for Šidák) show larger sensitivity for the same number of false positives. I have rephrased the sentence in the updated version of the manuscript.

> The manuscript is heavily written in first-person, maybe “depersonalize” it a little bit.

I’ve been back and forth about this. I want to be consistent with using “I” or “we” but not a mix

of both. Because this is a very personal work, I have decided to keep the first person.

> Figure 2 (a, b) has a space but (c,d) does not. The light blue font is a bit hard to read on Fig. 2.

Typo fixed. I have darken the blue font in Figure 2, and in related Figures S1, S2 and S3.

> A few noted minor grammar issues: e.g.
> “The Lancaster weights allow (one) to incorporate additional”
> “four aggregation[s] methods”
> “Two structure[s] ha(ve) been proposed”
> ”explore a[n] structural function”

Thanks for noticing the typos.

Reviewer #2

> Helix level conservation is a powerful way to build consensus structures (https://academic.oup.com/bioinformatics/article/22/24/2988/208807), does this metric aid R-scape in predicting new structures, either with cacofold or otherwise?

Yes, how to use helix-level covariation in the determination of a structure is the next application, but not for this manuscript. There is a certain circularity in using helix level covariation to predict a structure as (unlike base pair covariation), helix level covariation requires that a structure is proposed. Some more testing is required to incorporate helix level covariation into structure prediction.

Also, thanks for the reminder that the observation that covariation informed by stacking is more powerful than that observed for individual base pairs had been documented before. Noted in the manuscript.

> Now that R-scape is moving towards helix-level statistics, is there any chance covariation/consistency metrics (like the previously misused RAFS metric) can be reevaluated for their utility?

Not with this work. The misuse you refer to has to do with using a covariation measure incompatible with the null hypothesis. Aggregated p-values are based on the same null hypothesis, so nothing has changed regarding that. I haven’t added anything in the manuscript to describe this point which would take some time to explain properly, and I have described before (Gao et al. 2022).

> Didn’t proofread extensively but found minor typos, like “mayority” instead of “majority” on page 9 for example.

Typo fixed, and a few others. Thanks!

Reviewer #3

> My only suggestion would maybe suggest discussing why three of the four methods appear to perform very similarly (and extremely well) whereas sidak seems to underperform a bit, as this result raises some curiosity in a non-expert eye. While this is not necessary for the results presented to be convincing, it may help the discussion.

Thanks for the question. I've been thinking about this repeatedly, but I do not have a satisfactory answer yet. The fact that sidak aggregation performs similarly to 1-unaggregation makes sense as you are just looking at the minimum p-value in the helix. However, I was expecting a larger difference between the Fisher and the Lancaster or wFisher methods which take into account additional information in the form of covariation power, yet, all three methods perform very closely. This situation is similar to the aggregated p-values obtained for differential gene expression analysis [34]. In that case, the Lancaster method is weighted by transcript abundance, yet Fisher and Lancaster perform very close to each other as well. Lacking a good explanation, I have not added any comment in the manuscript.

I hope this revised manuscript addresses the reviewers' points, and I thank you again for the comments.

Sincerely,

Elena Rivas, Ph.D.
